# Chloroplast Genome Diversity and Molecular Evolution in Hypericaceae: New Insights from Three *Hypericum* Species

**DOI:** 10.3390/ijms26010323

**Published:** 2025-01-02

**Authors:** Kan Yan, Xin Lu, Wandi Li, Chao Sun, Xueqiong Zhou, Youyou Wang

**Affiliations:** 1 School of Biological and Pharmaceutical Engineering, Lanzhou Jiaotong University, Lanzhou 730070, China; lu_xin2022@163.com (X.L.); liwandi2022@163.com (W.L.); fxq092424@163.com (X.Z.); idafnj@163.com (Y.W.); 2 College of Agronomy, Gansu Agricultural University, Lanzhou 730070, China; sunc@gsau.edu.cn

**Keywords:** *Hypericum*, chloroplast genome, phylogenetic relationship, comparative genomics

## Abstract

The Hypericaceae family, comprising nine genera and over seven hundred species, includes *Hypericum* plants traditionally used for medicinal purposes. In this study, we performed high-throughput sequencing on three *Hypericum* species: *Hypericum acmosepalum*, *Hypericum addingtonii*, and *Hypericum beanii*, and conducted comparative genomic analyses with related species. The chloroplast genome sizes were 152,654 bp, 122,570 bp, and 137,652 bp, respectively, with an average GC content of 37.9%. All genomes showed a quadripartite structure, with significant variations in IR regions (3231–26,846 bp). The total number of genes ranged from 91 to 129. SSRs were predominantly located in the LSC region, with mononucleotide repeats being dominant. Comparative analysis identified several hotspot regions, including *accD*, *rpoC2*, *rpoB*, and *rpl22* in the LSC region and *matK*, *rpl32*, *rpl33*, and *rps4* in the SSC region. Nucleotide polymorphism analysis revealed eight highly variable regions and eleven gene loci, providing potential molecular markers for species identification. Phylogenetic analysis indicated that *Triadenum* and *Cratoxylum* are closely related to *Hypericum*, with *H. acmosepalum* and *H. beanii* being closest relatives and *Hypericum hookerianum* as their sister species. These findings provide molecular tools for species identification and insights for conservation strategies of medicinal *Hypericum* species.

## 1. Introduction

The Hypericaceae family, commonly known as the St. John’s wort family, comprises nine genera and over seven hundred species. It is divided into three tribes: Cratoxyleae, Hypericeae, and Vismieae [1]. However, the relationships within these tribes remain contentious. The largest genus within Hypericaceae is *Hypericum*, consisting of nearly five hundred herbaceous plants, shrubs, and small trees [2]. These plants are widely distributed globally, excluding Antarctica, with major concentrations in Eurasia and the Andes in South America. Significant numbers of species are also found in North America, Southeast Asia, and Africa [3]. Many *Hypericum* species have considerable medicinal value and have been traditionally used in various cultures to treat ailments such as eczema, menstrual disorders, burns, and digestive diseases [4,5,6]. The most renowned species, *Hypericum perforatum* (commonly known as St. John’s wort) [7], has been extensively studied and utilized for its antidepressant, antioxidant, antidiabetic, antihyperlipidemic, wound-healing, hepatoprotective, antiparkinsonian, renoprotective, anti-inflammatory, and antimicrobial properties [8,9,10] This species has brought significant economic value to the pharmaceutical and nutraceutical industries. Studies have shown that medicinal *Hypericum* species contain chemical compounds such as phloroglucinols, naphthodianthrones, xanthones, flavonoids, and terpenes [11,12], which are recognized for their biological activities.

Due to the significant medicinal value of *Hypericum* family members and their ability to adapt to various environmental conditions, they have become valuable resources for both agriculture and the pharmaceutical industry. Certain species of *Hypericum* plants have been cultivated commercially and at a large scale. However, the systematic taxonomy of *Hypericum* remains controversial due to the numerous species and their morphologically similar appearances. Accurate identification of *Hypericum* species with medicinal value is a key focus in plant breeding [13]. Understanding the chloroplast genomics of *Hypericum* species can significantly contribute to crop improvement strategies and the conservation of medicinal plants. This knowledge can lead to the development of more resilient and productive varieties, supporting sustainable agricultural practices. Moreover, insights gained from chloroplast genome analysis can inform conservation efforts for wild *Hypericum* populations, ensuring the preservation of genetic diversity crucial for future breeding programs and maintaining the availability of these important medicinal resources.

As early as the 18th century, *Hypericum* attracted the attention of taxonomists. Subsequently, Robson divided *Hypericum* into 36 sections, distinguishing species based on morphological traits such as habits, indumentum, glands, stems, leaves, sepals, and geographical distribution, proposing phylogenetic relationships among certain *Hypericum* species [14]. Further phylogenetic analysis using ITS sequences by Nürk et al. [2] revealed that Thornea is closely related to *Hypericum*, while *Triadenum* is nested within *Hypericum*. In recent years, the phylogenetic study of *Hypericum* has been a focal point for researchers. Early studies primarily relied on morphological characteristics, but with advancements in molecular biology techniques, molecular data have increasingly been applied to *Hypericum* phylogenetic research. Some researchers have used molecular markers such as nuclear genomes (nrDNA) and chloroplast genomes (cpDNA) to explore the phylogenetic relationships within *Hypericum*. For instance, Meseguer et al. [15]. conducted a comprehensive study of *Hypericum* phylogeny by integrating nuclear and chloroplast genome data. Despite these efforts, chloroplast genome studies on *Hypericum* remain relatively scarce, particularly lacking systematic comparative genomic analyses.

The chloroplast is a semi-autonomous organelle derived from ancient endosymbiotic cyanobacteria, responsible for photosynthesis in most higher plants [16,17]. It has the capability for self-replication, transcription, and translation. In most angiosperms, the chloroplast genome size ranges between 120 kb and 160 kb [18], featuring a typical quadripartite structure that includes a large single-copy region (LSC) and a small single-copy region (SSC) separated by a pair of inverted repeats (IRa and IRb) [19,20,21]. With the continuous development of sequencing technologies, chloroplast genome sequencing has become an effective tool for species identification [22]. The chloroplast genome is more conserved in terms of gene composition, structure, and gene number compared to nuclear and mitochondrial genomes [23]. Gene loss, IR expansion, structural rearrangements, and inversions frequently occur among different taxa [24,25], providing crucial insights into plant origins and evolutionary histories. Therefore, the chloroplast genome is a valuable source of information for phylogenetics, population genetics, and evolutionary studies.

In this study, high-throughput sequencing was performed for the first time on three *Hypericum* species: *Hypericum acmosepalum*, *Hypericum addingtonii*, and *Hypericum beanii*. These genomes were compared with those of eleven Hypericaceae species previously published in NCBI (National Center for Biotechnology Information), including *Hypericum ascyron* and *Hypericum hookerianum*, with two *Populus* species as outgroups. The objectives of this study were as follows: (1) to provide complete chloroplast genome data that reveal the chloroplast genome structural characteristics of these three species and reveal the chloroplast genome structural characteristics of these three species; (2) to develop potential molecular markers for the identification of *Hypericum* species; (3) to explore the phylogenetic positions of these three species within *Hypericum* and their relationships with subgenera in *Hypericum*; (4) to analyze evolutionary differences between *Hypericum* and its closely related genera, thereby providing data support for phylogenetic research and germplasm identification in *Hypericum*, and laying a foundation for understanding the evolutionary relationships within *Hypericum*.

## 2. Results

### 2.1. Genome Sequencing and Assembly

To elucidate the chloroplast genome sequence and structural characteristics of *Hypericum acmosepalum*, *Hypericum addingtonii*, and *Hypericum beanii*, sequencing was conducted using the NovaSeq6000 platform. The statistics of the obtained clean reads are presented in Table 1, showing that the sequencing error rate for the three *Hypericum* species was approximately 2.4%. The proportion of bases with Phred scores greater than 20 and 30 exceeded 95%, and the average length of sequencing fragments was around 147 bp.

**Figure 1:**
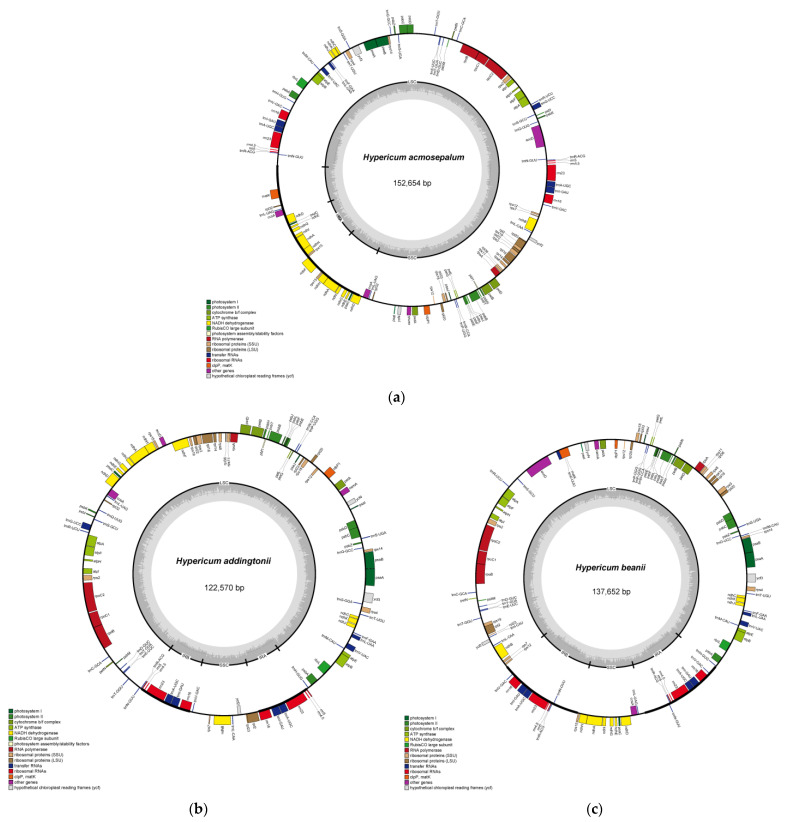
Chloroplast genomes of *H. acmosepalum* (**a**), *H. addingtonii* (**b**), and *H. beanie* (**c**). Genes located in the outer circle are transcribed counterclockwise, while those in the inner circle are transcribed clockwise. The colored bars represent different functional groups. The gray dashed area in the inner circle shows the GC content percentage of the respective genes. LSC, SSC, and IR represent large single-copy, small single-copy, and inverted repeat regions, respectively.

Using software such as NOVOPlasty and GetOrganelle, the sequencing files were assembled by splicing genome fragments and filling gaps, resulting in complete circular chloroplast genomes for *H. acmosepalum*, *H. addingtonii*, and *H. beanii*. Chloroplast genome maps were generated using OGDRAW (Figure 1). The total lengths of the genomes were 152,654 bp for *H. acmosepalum* (GC content ~37.55%), 122,570 bp for *H. addingtonii* (GC content ~38.01%), and 137,652 bp for *H. beanii* (GC content ~38.14%). Three genomes displayed a similar quadripartite structure and functional genes, though the sizes of the quadripartite regions varied, as did the genes transcribed in the inner and outer circles and the functions and positions of the genes.

### 2.2. Basic Structural Characteristics of the Chloroplast Genome

As shown in the chloroplast genome maps, the chloroplast genome structures of the three *Hypericum* species conform to the quadripartite structure. Comparing the basic structural data of the chloroplast genomes of 14 Hypericaceae species and two outgroup species (Table 2), the total length of these chloroplast genomes ranged from 122,570 bp to 167,693 bp, with a difference of 45,123 bp between the longest and shortest genomes. The total number of genes in the 16 genomes ranged from 91 to 129, with the total gene count in *Hypericum* species fluctuating between 109 and 127, while *Cratoxylum* species had a stable gene count of around 128. The average length of the chloroplast genomes of the 16 species was 151,009.50 bp. *H. addingtonii* had the shortest genome length, followed by *H. beanii*, both significantly shorter than the average chloroplast genome length. *Triadenum breviflorum* had the longest genome length.

In all 16 species, the chloroplast genomes contained four characteristic regions. The LSC length ranged from 84,618 bp (*Populus alba*) to 126,934 bp (*H. acmosepalum*), the SSC length ranged from 3044 bp (*H. acmosepalum*) to 18,971 bp (*Cratoxylum maingayi*), and these two regions were separated by two IR regions (IRa and IRb) ranging from 3231 bp (*Hypericum hookerianum*) to 35,620 bp (*Triadenum breviflorum*). Significant structural differences were observed among the chloroplast genomes of different genera, such as *Cratoxylum sumatranum*, *Cratoxylum pruniflorum*, *Cratoxylum maingayi*, and *Cratoxylum formosum*, which had stable LSC, SSC, and IR regions sizes around 86,037 bp, 26,124 bp, and 18,811 bp, respectively. In contrast, the LSC length of the seven *Hypericum* chloroplast genomes fluctuated between 85,268 bp and 126,934 bp, SSC length between 3044 bp and 11,185 bp, and IR length between 3231 bp and 35,620 bp, with notable variation in IR region sizes. Notably, the chloroplast genome of *H. acmosepalum* exhibited the most unique structure among the seven *Hypericum* species, with the largest LSC region (126,934 bp) and the smallest SSC region (3044 bp). Moreover, the total GC content of the 16 chloroplast genomes ranged from 36.15% (*Cratoxylum arborescens*) to 38.14% (*H. beanii*), with a difference of 1.99%. The average total GC content was approximately 37.04%. *Hypericum* species had an average GC content of 37.76%, while *Cratoxylum* species had an average GC content of 36.23%, indicating higher GC content in *Hypericum* species and significant variation in IR-region GC content. Further analysis suggested that structural changes in the *Hypericum* chloroplast genomes might be related to base substitution rates.

As shown in Table 2, *Hypericum* species exhibited more notable fluctuations in gene numbers compared to *Cratoxylum* species, attributed to the instability of protein-coding genes and tRNA genes in *Hypericum*. In *Hypericum* species, the total gene count ranged from 109 to 127, protein-coding genes (PCGs) from 71 to 86, and tRNA genes from 29 to 37, with significant variation in the number of multi-copy genes within the PCGs. In contrast, *Cratoxylum* species had stable chloroplast genome total gene counts of 127 or 128, stable protein-coding gene counts of 82 or 83, stable tRNA counts of 37, and stable rRNA counts of eight, with minimal fluctuations in the number of multi-copy genes.The functional gene tables of the genomes of three *Hypericum* plants (*Hypericum acmosepalum*, *Hypericum addingtonii*, and *Hypericum beanie*) are shown in Supplementary Table (Table S1). The genes of three species of *Hypericum* plants can be broadly categorized into three major types: self-replication genes, photosynthesis-related genes, and other genes. Table S1 can visually represent the variations in the types and quantities of genes among different species. For instance, the chloroplast genome of *H. acmosepalum* lacks the *matK* gene, which is crucial for the functional expression of chloroplast genes. The chloroplast genome of *H. addingtonii* does not contain the *ndhH* gene, which is involved in the composition of dehydrogenase and plays a role in stress response [26].

### 2.3. Simple Sequence Repeats Analysis

Simple sequence repeats (SSRs) are efficient molecular markers characterized by high abundance, good reproducibility, co-dominant inheritance, uniparental inheritance, and relative conservation, making them ideal for species identification and genetic variation assessment at both population and individual levels [27,28].

**Figure 2:**
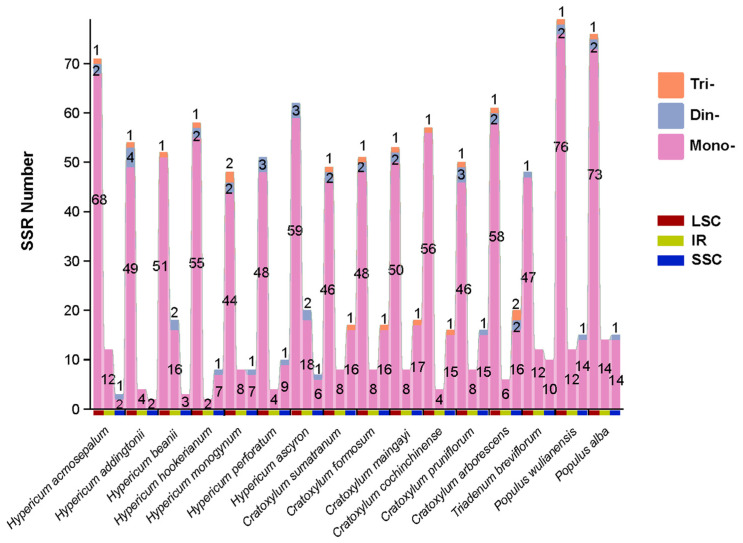
Distribution of SSR numbers in 16 species. The horizontal axis represents different species, and the vertical axis represents the number of SSRs. Red blocks represent the LSC region, green blocks represent the IR region, and blue blocks represent the SSC region. Mono-, mononucleotide repeats; Din-, dinucleotide repeats; Tri-, trinucleotide repeats; Tetra-, tetranucleotide repeats.

Using MISA technology, we analyzed the number and distribution of SSRs in the chloroplast genomes of 16 species (Figure 2). The results showed that the numbers of SSRs in *H. acmosepalum*, *H. addingtonii*, and *H. beanii* were 86, 60, and 73, respectively. Among the 16 species, SSRs primarily consisted of three types: mononucleotide repeats, dinucleotide repeats, and trinucleotide repeats, with only *H. ascyron*, *H. perforatum*, and *T. breviflorum* lacking trinucleotide repeats in their chloroplast genomes. SSRs were widely distributed within the quadripartite structure of the chloroplast genome, predominantly in the LSC region, with mononucleotide repeats being the main SSR type. In *Hypericum* species, the LSC region was the primary distribution area for SSRs, with trinucleotide repeats only appearing in the LSC region. Unlike *Hypericum* species, *Cratoxylum* species also had SSRs in the SSC region, with a full range of SSR types, though predominantly mononucleotide and trinucleotide repeats. Notably, differences in SSR distribution among *Hypericum* species were mainly concentrated in the IR region. The specific distribution locations and areas of SSRs in the chloroplast genomes of *H. acmosepalum*, *H. addingtonii*, and *H. beanii* are provided in the Supplementary Table (Table S2), indicating that most SSRs were located in intergenic regions, with a few in intron regions. The genes containing SSRs primarily include *trnG-UCC*, *rpoC1*, *rpl32*, *ndhA*, *petB*, *atpF*, *ycf3*, *rps19*, *rpl2*, *ndhH*, *rps12*, *trnA-UGC*, *rpoC2*, and *psbF*, among others.

**Figure 3:**
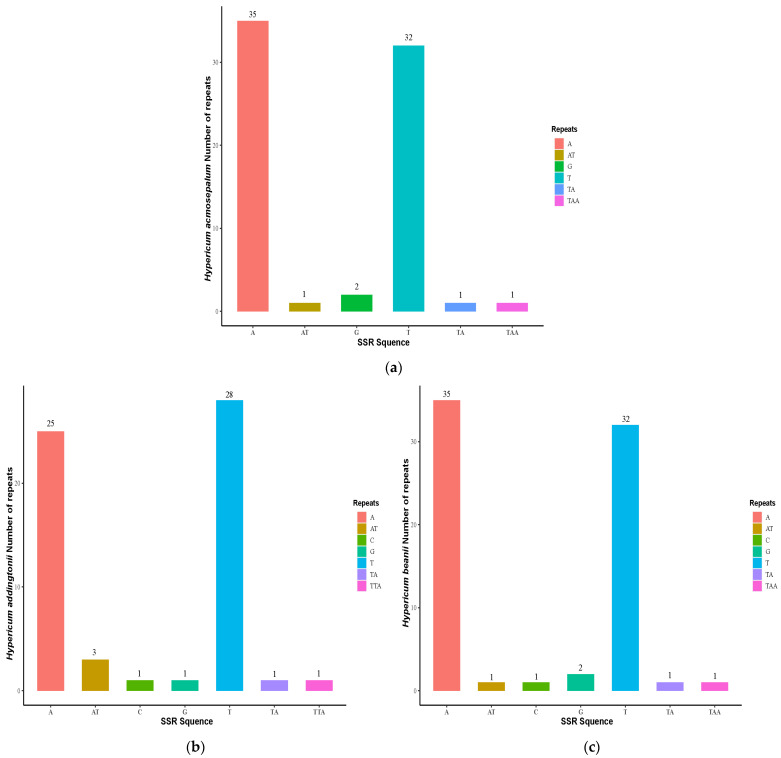
Statistics of SSR base repeat types in *H. acmosepalum* (**a**), *H. addingtonii* (**b**), and *H. beanie* (**c**). The horizontal axis represents SSR base repeat types, and the vertical axis represents SSR repeat quantities.

Figure 3 shows the base repeat types and repeat quantities of SSRs in the chloroplast genomes of *H. acmosepalum*, *H. addingtonii*, and *H. beanii*. The results indicate that in the three sequenced *Hypericum* species, SSRs mainly consisted of A and T bases, which accounted for a high proportion of all SSR sequences (93.06% in *H. acmosepalum*, 88.33% in *H. addingtonii*, and 91.78% in *H. beanii*). Among them, A base repeats were more frequent in the chloroplast genomes of *H. acmosepalum* and *H. beanii*, while T base repeats were more frequent in the chloroplast genome of *H. addingtonii*.

### 2.4. Analysis of Long-Repeat Sequences

**Figure 4:**
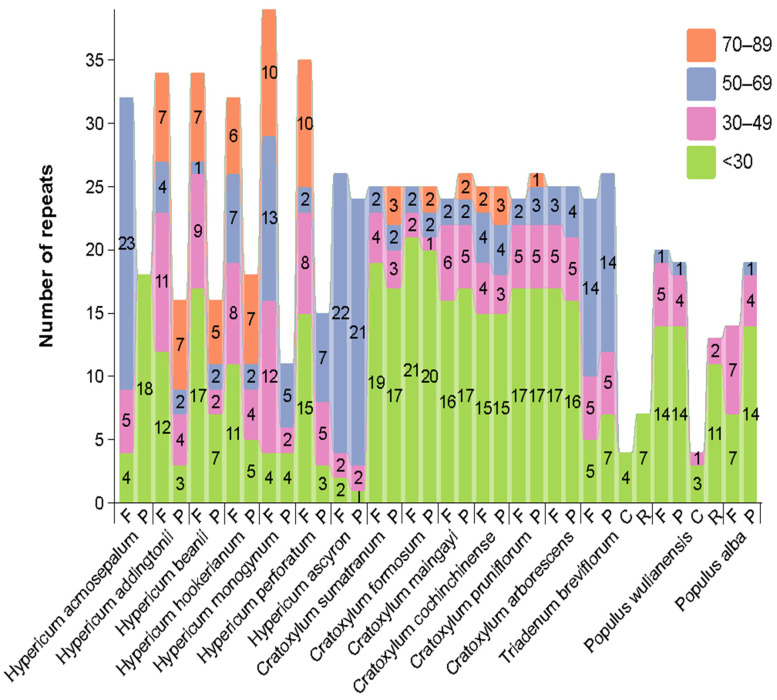
Analysis of long-repeat sequences in 16 different chloroplast genomes. The *x*-axis represents long-repeat types, and the *y*-axis represents the quantity of the corresponding long-repeat types. F: forward; R: reverse; C: complement; P: palindromic. Different colors represent different sizes of long-repeat sequences.

Long-repeat sequences can be categorized by length into five types (<30, 30–49, 50–69, 70–89, ≥90 bp) and by type into forward (F), reverse (R), complement (C), and palindromic (P) repeats. The long-repeat sequences in the complete chloroplast genomes of 16 species were analyzed (Figure 4). In terms of repeat sequence types, Hypericaceae plants had only forward and palindromic repeats, with no complement or reverse repeats. For *Cratoxylum* plants, the numbers of forward and palindromic repeats were roughly similar, with a ratio close to 1:1. However, for *Hypericum* plants, the number of forward repeats was significantly greater than that of palindromic repeats, showing a 2:1 trend.

Regarding the length of long-repeat sequences, all plants had sequences with lengths of <30 bp, 30–49 bp, and 50–69 bp. Only *H. acmosepalum*, *H. ascyron*, *C. arborescens*, *T. breviflorum*, and the two outgroup plants lacked sequences of 70–89 bp. For *Hypericum* plants, long-repeat sequences were mainly large fragments (sequence sizes greater than 30 bp), similar to *T. breviflorum*, while *Cratoxylum* species predominantly had small-fragment sequences (sequence sizes less than 30 bp). Notably, the large-fragment long repeats in *Hypericum* were mainly concentrated in forward repeats, possibly related to gene rearrangements.

### 2.5. Codon Usage Preference Analysis

Codon usage preference refers to the non-uniform usage of synonymous codons encoding the same amino acid. This phenomenon results from the combined effects of mechanisms such as mutation and selection over long periods of evolution. In organisms, genes with high expression levels tend to use certain codons more frequently to enhance accuracy and efficiency. Therefore, codon usage preference analysis can help infer the degree of gene expression selection in different species [29].

**Figure 5:**
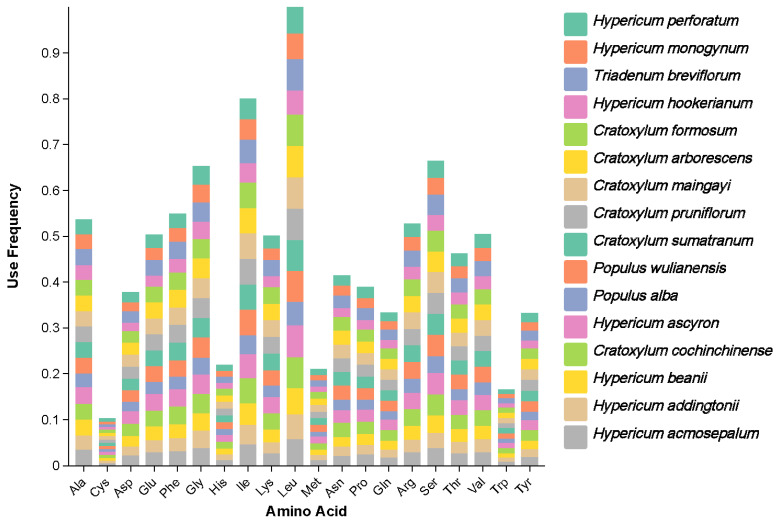
Frequency of amino acids in the codon coding regions of the chloroplast genomes of 16 species. Frequency: The relative frequency of codons (amino acids) used in different species relative to leucine (Leu).

**Figure 6:**
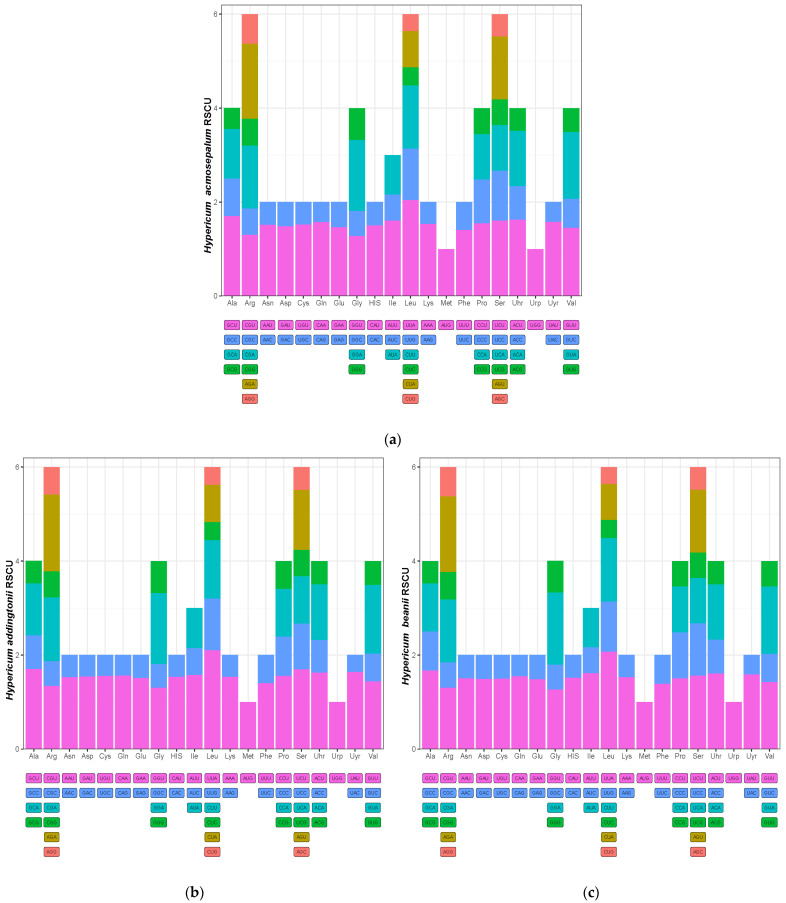
Relative synonymous codon usage (RSCU) of *H. acmosepalum* (**a**), *H. addingtonii* (**b**), and *H. beanii* (**c**). The horizontal axis represents amino acids, and the vertical axis represents RSCU values. Differently colored blocks represent different codons.

Figure 5 shows the codon usage frequencies of different amino acids relative to leucine (Leu) in 16 plant species. Among them, the codons encoding Leu were the most frequently used, followed by those encoding isoleucine (Ile) and serine (Ser). Figure 6 illustrates the relative synonymous codon usage (RSCU) of *H. acmosepalum*, *H. addingtonii*, and *H. beanii*. According to the data in Figure 6, most amino acids showed codon preference (RSCU value greater than 1) in the three sequenced plants. The codons preferred for Leu, Ile, and Ser were TTA, ATT, and TCT, respectively.

**Figure 7:**
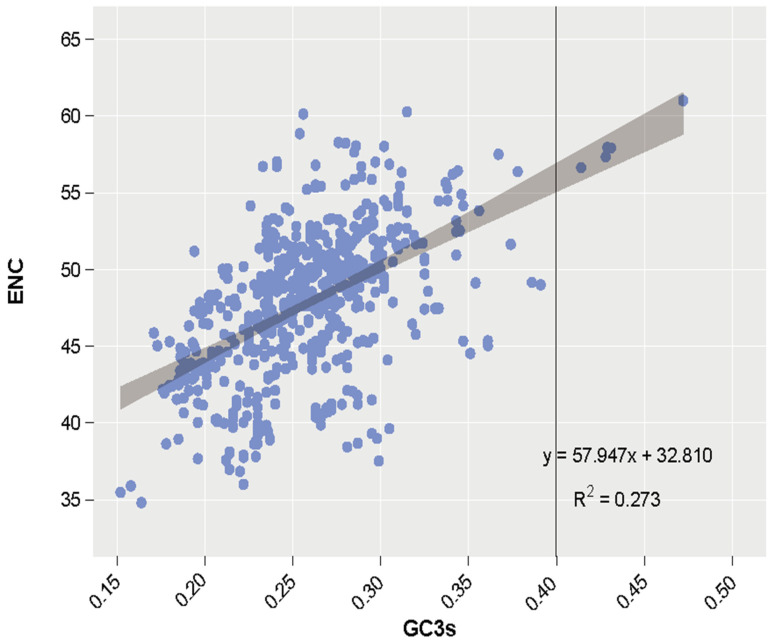
ENC-plot analysis of 16 species. The horizontal axis represents the GC content at the third codon position (GC3s), excluding tryptophan, methionine, and stop codons. The vertical axis represents the effective number of codons.

ENC-plot analysis is commonly used to display the factors influencing codon usage preference in genomes. A higher ENC value indicates more uniform codon usage. Gene points falling above the diagonal line suggest that factors other than GC3s (GC content at the third position of synonymous codons) are influencing codon usage preference [30]. According to the ENC plot (Figure 7), coding genes exhibited certain codon preferences, with a stronger inclination towards A and T bases. The points in the graph mainly hovered near the expected curve, indicating that the formation of codon usage preference is primarily influenced by base mutations.

### 2.6. Expansion and Contraction of Boundary Regions

**Figure 8:**
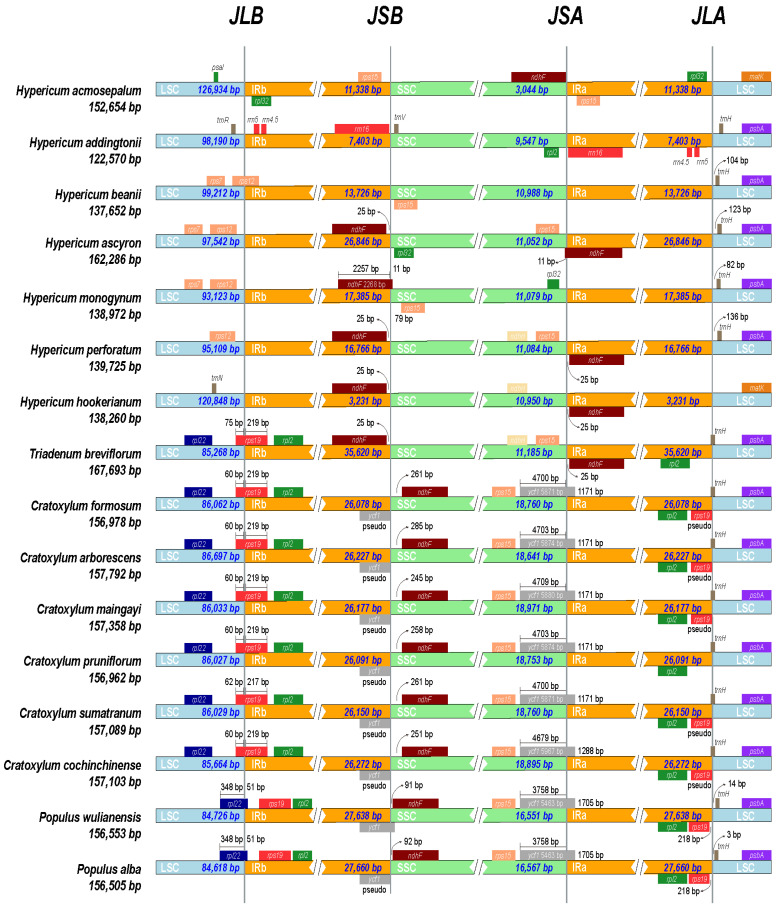
Comparison of LSC, IR, and SSC boundary regions in 16 chloroplast genomes. (Light blue, yellow, and green blocks represent LSC, IR, and SSC regions, respectively. JSA: junction of SSC and IRA; JLB: junction of LSC and IRB; JSB: junction of SSC and IRB. The color boxes above or below the main line represent genes adjacent to the boundaries.)

The expansion and contraction of inverted repeats (IRs) can lead to structural changes in the chloroplast genome, such as variations in the number of gene copies or the production of pseudogenes in boundary regions, making them significant factors in chloroplast genome variation [31]. The specific positions and lengths of these regions are crucial evolutionary characteristics between species. Therefore, we compared the boundaries and boundary genes of 16 species, including 14 Hypericaceae species and two outgroup species, and analyzed the expansion and contraction of the junction regions in detail (Figure 8).

Alignment analysis of the chloroplast genomes of 16 species revealed significant structural differences. The genome structure of *Cratoxylum* was relatively stable, while the genome structure of *Hypericum* showed more pronounced differences. For the chloroplast genomes of seven *Hypericum* species, the LSC–IRb boundary rarely overlapped with genes, except in *H. beanii*, where it was located within the *rps12* gene. The IRb–SSC boundary deviated by 25 bp from the *ndhF* gene in most *Hypericum* chloroplast genomes. In contrast, the *rps15* and *rrn16* genes corresponded to the *ndhF* gene position in the chloroplast genomes of *H. acmosepalum* and *H. addingtonii*, while the *ndhF* gene was absent in the *H. beanii* chloroplast genome. The SSC–IRa boundary in *Hypericum* species and *Triadenum breviflorum*’s chloroplast genomes almost did not intersect with genes, except in *H. ascyron*, where the *ndhF* gene spanned the boundary by 11 bp.

Regarding gene arrangement, structure, and quantity, *Cratoxylum* plants were generally similar to outgroup plants but differed significantly from *Hypericum* plants. Notably, *T. breviflorum*’s chloroplast genome structure partially resembled that of *Hypericum* and *Cratoxylum* chloroplast genomes, appearing as a mosaic of chloroplast genomes from different genera. Additionally, there were differences in gene arrangement, structure, and quantity among the chloroplast genomes of *Hypericum* species. It can be preliminarily inferred that *Hypericum* plants underwent more nucleotide variations during evolution. Further analysis, as shown in the figure, indicates significant variation in the IR regions of *Hypericum* chloroplast genomes, with sizes ranging from 3231 bp to 26,846 bp, all dissimilar. This suggests that changes in the IRs region are the main reasons for the structural differences in *Hypericum* chloroplast genomes.

### 2.7. Sequence Diversity Analysis of the Chloroplast Genome

**Figure 9:**
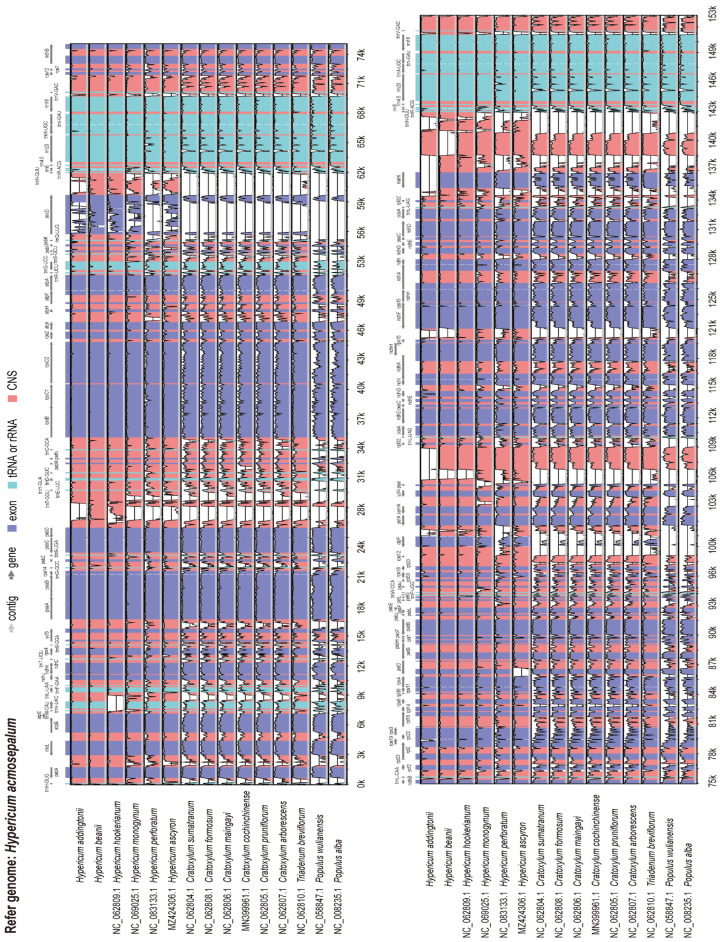
mVISTA comparison of the chloroplast genomes of 16 plant species. The *x*-axis represents nucleotide sequences, and the *y*-axis represents sequence homology percentages ranging from 50% to 100%. Conserved regions are highlighted below the curve, with purple indicating conserved exons and gray arrows indicating gene transcription directions. Red bars represent non-coding sequences (CNS), and light green bars represent tRNA or rRNA.

The mVISTA whole-genome sequence alignment tool was employed to analyze the sequence similarity of the chloroplast genomes of 16 plant species, visualizing the homology among them. The results indicated that the chloroplast genomes of *Hypericum* species exhibited high sequence similarity (Figure 9), suggesting relatively conserved genome structures at the gene sequence level. Similarly, the chloroplast genomes of *Cratoxylum* species also showed high sequence similarity but displayed significant differences when compared to *Hypericum* species. Notably, the chloroplast genome sequences of *Cratoxylum* species were highly similar to those of the outgroup species. Further analysis revealed that the IR regions of the 16 species exhibited less variation compared to the LSC and SSC regions. The chloroplast genome structure of *Hypericum* species was found to be less stable than that of *Cratoxylum* species, with significant differences between the genome structures of the two genera. Therefore, identifying highly variable nucleotide regions in the 16 chloroplast genomes is crucial for applications such as species phylogeny, molecular identification, and molecular barcoding. For example, in the LSC region, highly variable non-coding sequences included *psbA-rbcL*, *trnV-UAC-trnL-UAA*, *ycf3-psaA*, *trnG-GCC-psbZ*, *psbD-trnT-GGU*, *atpI-atpH*, *accD-trnN-GUU*, and *trnT-UGU-rps4*. Highly variable exonic sequences included the *accD*, *rpoC2*, and *rpoB* genes. In the SSC region, highly variable non-coding sequences included *matK-trnN-GUU*, *psaI-rpl32*, *rpl32-matK*, *trnL-UAG-rpl32*, *rps15-ndhF*, *ndhD-ccsA*, and *ndhG-ndhI*. Highly variable exonic sequences included the *rpl32*, *rps4*, *rps15*, *ccsA*, *clpP*, *rpl33*, *rpl22*, *ycf2*, and *matK* genes. For *Hypericum* species, the non-coding regions exhibited significant sequence differences, with many highly variable regions, while the exonic gene regions were relatively stable, with most of the highly variable regions concentrated in the SSC region. These highly variable regions could serve as potential molecular markers for species identification and germplasm resource development in *Hypericum*.

### 2.8. Nucleotide Polymorphism Analysis

**Figure 10:**
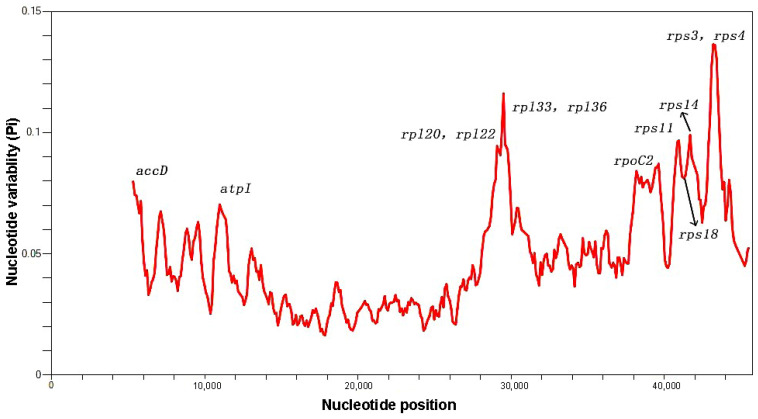
Sliding window analysis of nucleotide diversity (π) in the chloroplast genomes of 16 species. The *x*-axis represents the midpoint position of the window, and the *y*-axis represents nucleotide diversity (π) for each window.

Nucleotide polymorphism analysis identifies highly variable sites across the chloroplast genome, which is crucial for species identification and phylogenetic analysis. In this study, sliding window analysis was used to conduct nucleotide diversity (Pi) tests on 16 species, including Hypericaceae and outgroup plants, resulting in the identification of eight highly variable regions with 11 gene loci (Figure 10). The peaks in the line chart correspond to nucleotide polymorphism sites with respective Pi values. For instance, the Pi values for the *rpl20* and *rpl22* loci were 0.09245, and for the *rpl33* and *rpl36* loci were 0.1161, while the Pi values for the *rps3* and *rps4* loci were 0.13615. The highest nucleotide diversity was observed in the *rps3* and *rps4* regions (Pi value of 0.13615), followed by the *rpl33* and *rpl36* regions and the *rps14* region, all with values greater than 0.06.

### 2.9. Phylogenetic Analysis

**Figure 11:**
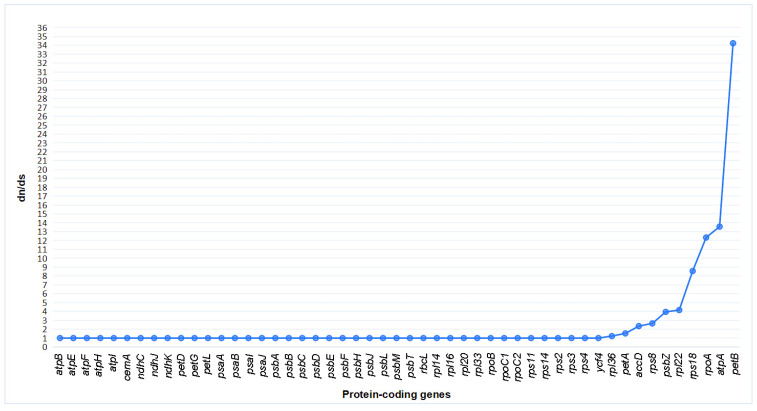
Selection pressure analysis of 53 shared protein-coding genes in the chloroplast genomes of 16 species.

**Figure 12:**
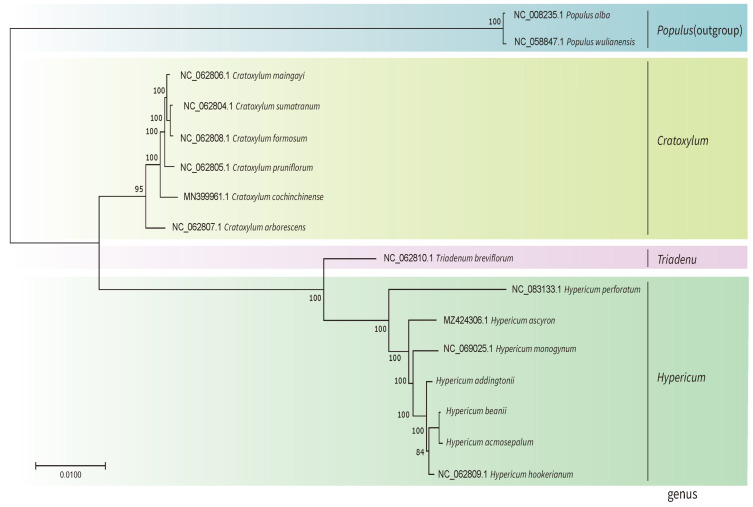
Phylogenetic relationships inferred by maximum likelihood (ML) for 16 species. The ML tree based on 41 protein-coding genes (CDS) is marked in red.

To analyze the phylogenetic relationships of the three *Hypericum* species, a phylogenetic tree of 16 species was constructed using the maximum likelihood (ML) method, assessing the phylogenetic relationships and positions of *H. acmosepalum*, *H. addingtonii*, and *H. beanii*. dN/dS analysis was performed on 53 protein-coding genes of the 16 species, and the ratio of synonymous (Ks) to non-synonymous (Ka) substitution rates (ω = Ka/Ks) was calculated using the CODEML tool in PAML. In Ka/Ks analysis, ω > 1 indicates positive selection; ω = 1 indicates neutral selection; ω < 1 indicates purifying selection (indicating that nucleotide changes are naturally occurring without selective pressure). As shown in Figure 11, we calculated the ω values of 53 protein-coding genes and selected 41 genes with ω values of 1 for constructing a phylogenetic tree using their CDS sequences (Figure 12). According to the molecular clock theory, nucleotide evolution rates among different lineages are roughly constant and proportional to time. Constructing an evolutionary tree using these sequences can visually represent species evolution, phylogenetic relationships, and divergence times.

The results showed high support values for all nodes in the ML tree topology. The phylogenetic tree indicated that *Triadenum* species shared a common ancestor with *Hypericum* species, suggesting a close relationship between *Triadenum* and *Hypericum*. In contrast, *Cratoxylum* species were more distantly related to *Hypericum*. In the *Hypericum* branch, *H. hookerianum*, *H. acmosepalum*, *H. beanii*, and *H. addingtonii* were closely related, with *H. acmosepalum* and *H. beanii* being the closest (support value of 100%). They also shared a common ancestor with *H. hookerianum* (support value of 84%). Additionally, the branch lengths in the phylogenetic tree for *Hypericum* species were longer compared to those of *Cratoxylum* species, indicating a higher degree of genetic variation among *Hypericum* species, which may be related to *Hypericum*’s strong adaptability.

**Figure 13:**
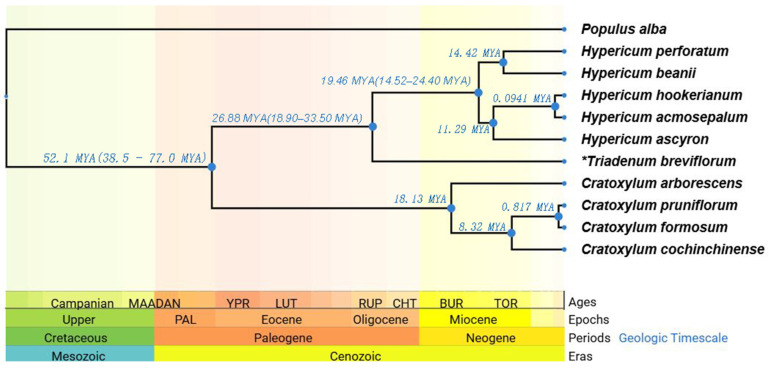
Divergence time tree for selected Hypericaceae plants. The time tree shows species divergence times, with blue font indicating the divergence time at each node and numbers in parentheses representing the range of divergence times. The average value is taken to represent divergence time, and the lower area indicates the period of divergence time. Data are based–on highly reliable values (>95%) from past literature. In geographic timescale, different color blocks correspond to the branches of time tree, “*” indicates that this plant is distinct from other genera.

Moreover, this study utilized data from the TimeTree online database, which constructs divergence time trees based on extensive published literature data (Figure 13). These data allow us to infer divergence times and phylogenetic relationships among different genera, explaining the structural differences in their chloroplast genomes. The divergence time tree indicated that *Hypericum* and *Triadenum* diverged from their common ancestor around 26.88 MYA (18.90–33.50 MYA), a time point calibrated through fossil records and molecular data [32,33,34]. This divergence time provides key evidence for the evolutionary relationship between the two genera. *Cratoxylum* diverged around 52.1 MYA, which may explain the similarities and differences in the chloroplast genome structures of the three genera. Based on fossil records and molecular clock calibration results, 52.1 MYA (38.5–77.0 MYA) is likely the earliest divergence time for Hypericaceae plants [15,35,36,37]. Within this time frame, the common ancestor of Hypericaceae species evolved for various reasons, leading to the differentiation of *Cratoxylum* species, which gradually increased over time. This reflects the unique position of *Cratoxylum* in the evolutionary history of Hypericaceae and explains the stability of its chloroplast genome structure. From 19.46 MYA (14.52–24.40 MYA), species such as *H. hookerianum*, *H. acmosepalum*, and *H. beanii* gradually evolved. Among them, *H. perforatum* is the first species to diverge within *Hypericum*, followed by *H. ascyron*, enriching the *Hypericum* family [15,36,38,39]. The phylogenetic tree clarified the evolutionary positions of *H. acmosepalum*, *H. addingtonii*, and *H. beanii* within *Hypericum* species and provided accurate data illustrating the phylogenetic relationships among Hypericaceae species.

## 3. Discussion

### 3.1. Structural Variations in the Chloroplast Genomes of Hypericum Species

In this study, we successfully elucidated the complete chloroplast genome sequences of *Hypericum acmosepalum*, *Hypericum addingtonii*, and *Hypericum beanii* and conducted an in-depth comparative genomic analysis. The results showed that the average size of the chloroplast genomes of these three species was 137,625 bp, with an average GC content of 37.9%, which is highly consistent with the structural characteristics, gene content, and arrangement of typical angiosperm chloroplast genomes. However, significant differences were observed in the chloroplast genomes among *Hypericum* species compared to other closely related species. By comparing the chloroplast genomes of seven *Hypericum* species, six *Cratoxylum* species, and one *Triadenum* species, we found that the quadripartite structure size varied significantly in *Hypericum*, especially in the IR region, spanning from 3231 bp to 26,846 bp, and that rearrangements and deletions accompany genes such as *ndhF*, *rps19*, and *rpl2*, suggesting instability in the chloroplast genome structure of *Hypericum* species. Xinyu Liu et al. demonstrated that sequence rearrangements and deletions in the genome are among the reasons for variation in genome size [7], which is consistent with our results. This genome compression may enhance replication efficiency and reduce energy consumption, potentially providing advantages in their respective environments.

To investigate the causes of structural differences in chloroplast genomes, we analyzed long-repeat sequences. The results showed that, unlike the outgroup plants, Hypericaceae plants only had forward and palindromic repeats, with no complement or reverse repeats. *Cratoxylum* plants had similar numbers of forward and palindromic repeats, with a roughly 1:1 ratio and predominantly small-fragment repeats less than 30 bp. In contrast, *Hypericum* had more large-fragment repeats, mainly concentrated in forward repeats, with a forward-to-palindromic repeat ratio trending towards 2:1. Claude et al. suggested that repeat sequences might contribute to rearrangements, which are considered one of the causes of gene structure variations [40]. Therefore, the high content of long-repeat sequences in *Hypericum* might indicate a higher tendency for genome rearrangements, potentially explaining the structural variations in *Hypericum* chloroplast genomes.

Furthermore, the contraction and expansion of the IR region are considered important mechanisms for chloroplast genome structural variation and evolution [24,41,42]. Comparative analysis of the chloroplast genomes of 16 species revealed significant structural differences, with *Cratoxylum* exhibiting relatively stable genome structures and *Hypericum* showing more pronounced differences. In the chloroplast genomes of the seven *Hypericum* species, the boundaries mostly did not overlap with genes, except for genes located near the boundaries. For example, the IRb–SSC boundary deviated by 25 bp from the *ndhF* gene in most *Hypericum* chloroplast genomes. Notably, the *rps15* and *rrn16* genes, instead of the *ndhF* gene, were present at the corresponding position in the chloroplast genomes of *H. acmosepalum* and *H. addingtonii*, while the *ndhF* gene was absent in *H. beanii*. In addition, the substituted *ndhF* genes in *H. acmosepalum* and *H. addingtonii* are found in the SSC and LSC regions, respectively. This suggests that structural changes in the chloroplast genome might be related to variations in the IR region. In the chloroplast genomes of *Hypericum*, the size of the IR region varied significantly, ranging from 3231 bp to 26,846 bp, with dissimilar sizes in the IR regions of the seven *Hypericum* species. The data testified to the above view.

Using CodonW software, we analyzed the codon usage preferences of protein-coding genes in the chloroplast genomes of 16 species. The results indicated that 64 codons were utilized, with leucine (Leu) being the most frequently encoded amino acid, followed by isoleucine (Ile) and serine (Ser). The most commonly used codons for these amino acids were TTA, ATT, and TCT, respectively. Previous studies have shown that GC content is closely related to mutational pressure or natural selection, and that mutational pressure acts on GC3s [43]. According to the ENC plot of the 16 species, most genes exhibited specific codon preferences, with GC3s values primarily around 0.25–0.30, suggesting a stronger preference for A and T bases in coding genes. Most genes clustered near the expected curve, implying that the codon usage bias is predominantly influenced by mutational pressure resulting from environmental stress, consistent with Claude et al.’s findings [40]. This suggests that high base mutation rates may play a significant role in the evolution of *Hypericum* species, while differences in codon usage frequencies among species could be related to evolutionary status, living environments, and base composition [7]. Furthermore, SSRs exhibit a preference for A and T bases as well. The two hydrogen bonds formed by A/T base pairing are more susceptible to breakage compared to the three hydrogen bonds formed by G/C base pairing. Consequently, the preference for A and T bases in the chloroplast genome of *Hypericum* species may be a contributing factor to their strong environmental adaptability and significant genomic structural variations. The formation of the preference for A and T bases may be related to the accumulation of G/C→A/T mutations during DNA replication and repair processes, as well as facilitating the unwinding of the strand during transcription and replication [44]. However, further research is needed to elucidate the underlying mechanisms.

### 3.2. Molecular Marker Development and Species Identification of Hypericum

For *Hypericum* species, whether for studying plant evolutionary relationships or for medicinal production, developing molecular markers and conducting species identification are essential. High-efficiency molecular markers can be utilized for species identification of *Hypericum* plants with medicinal value at any stage of preservation, enabling the distinction between morphologically similar species with low pharmaceutical efficacy and facilitating the large-scale cultivation of *Hypericum* medicinal plants [45]. Among them, the characteristics of SSR (simple sequence repeat) molecular markers are conducive to distinguishing between homozygotes and heterozygotes. When traits of interest are associated with genes, molecular markers can be used to select appropriate parents, facilitating efficient plant breeding based on genetic information [46,47,48,49]. This study analyzed the number and repeat types of SSRs in the chloroplast genomes of 16 species. The results showed that SSRs in *Hypericum* chloroplast genomes were primarily distributed in the LSC region and mainly consisted of mononucleotide repeats. Subsequently, the types, quantities, and locations of SSRs in *H. acmosepalum*, *H. addingtonii*, and *H. beanii* were statistically analyzed. The findings indicated that SSRs exhibited rich polymorphism among closely related species, predominantly located in intergenic regions and intron sequences, covering genes such as *ndhA*, *petB*, *atpF*, *ycf3*, *rps19*, *rpl2*, *ndhH*, and *rps12*. Notably, the *ndhH* gene is associated with stress responses [50]. Studies have shown that the IR region is highly variable in the chloroplast genome. The differences observed in genes such as *rps19* and *rpl2* in *H. acmosepalum*, *H. addingtonii*, and *H. beanii*, compared to other *Hypericum* species within the chloroplast genome IR regions, suggest that SSR polymorphism may be related to the expansion and contraction of its boundaries, which can alter the distribution positions of some SSR sites [51]. This provides data support for identifying potential molecular markers for species identification and molecular breeding.

To understand the evolutionary characteristics of *Hypericum* chloroplast genomes, we conducted whole-genome comparisons using the mVISTA software. The results revealed that the chloroplast genomes of Hypericaceae species were relatively conserved within the same genus, while differences were more pronounced among different genera. Based on the comparison results, we identified several highly variable regions, such as the *accD*, *rpl22*, and *rpoB* genes in the LSC region, and the *matK*, *rpl32*, *rpl22*, *rpl33*, and *rps4* genes in the SSC region. Further analysis using DnaSP6 software identified highly variable sites among the 16 species, including Hypericaceae and outgroup plants, revealing 11 gene loci: *accD*, *atpI*, *rpl20*, *rpl22*, *rpl33*, *rpl36*, *rpoC2*, *rps11*, *rps14*, *rps33*, and *rps4*. These loci exhibited high nucleotide diversity indices among the common coding genes of the 16 species. These variable regions are expected to provide potential molecular markers or DNA barcodes for *Hypericum* species identification, germplasm resource development, and elucidation of the evolutionary relationships of Hypericaceae.

### 3.3. Evolutionary Relationships of Hypericum Species

Regarding the phylogenetic relationships of *Hypericum*, this study found that *Hypericum monogynum* had the shortest branch length in the phylogenetic tree, indicating it diverged from the common ancestor most recently, followed by *H. addingtonii*. *Hypericum hookerianum*, *H. beanii*, and *H. acmosepalum* followed in divergence. *H. hookerianum* and *H. acmosepalum* were closely related, with *H. acmosepalum* being more closely related to *H. beanii*, supported by a value of 100%, indicating they are the closest relatives.

In 2012, Robson et al. morphologically classified *Triadenum* as a genus adjacent but distinct from *Hypericum*. In 2013, Nürk et al. [2] conducted a phylogenetic analysis of Hypericaceae using ITS sequences of nuclear DNA and classified *Triadenum* within *Hypericum*, which led to significant disagreement. In this study, phylogenetic analyses of the chloroplast genomes of *Hypericum*, *Triadenum*, and *Cratoxylum* were performed. The results showed that although the evolutionary position of *Triadenum* is close to *Hypericum*, Figure 8 indicates that the chloroplast gene structure of *Triadenum* is partially similar to that of *Hypericum* (SSC region and part of the IR region) and partially similar to that of *Cratoxylum* (LSC region and part of the IR region). Furthermore, the divergence time analysis in Figure 13 reveals that *Triadenum* diverged earlier than *Hypericum*, roughly by 7.42 million years ago (MYA). These data suggest a high probability that *Triadenum* is a genus adjacent but distinct from *Hypericum*. These findings support the independent divergent status of *Triadenum* at the molecular level. Additionally, the phylogenetic tree constructed by Nürk et al. using ITS sequences provided unclear phylogenetic relationships among *H. beanii*, *H. hookerianum*, and *H. acmosepalum*, with support values below 70%, indicating low data reliability. In contrast, chloroplast genomes, characterized by simple structures, conserved sequences, and moderate evolutionary rates, offer higher resolution and reliability for constructing phylogenetic trees [31]. Figure 12 clarifies the phylogenetic relationships among *H. beanii*, *H. hookerianum*, and *H. acmosepalum*, with a support value of 84%, indicating reliable data. *H. acmosepalum* and *H. beanii* are the closest relatives, while *H. hookerianum* is the sister species to both *H. acmosepalum* and *H. beanii*. *H. addingtonii* shares a common ancestor with *H. acmosepalum*, *H. beanii*, and *H. hookerianum*, supported by 100%, 84%, and 100%, respectively. Notably, in our study, the branch length of *H. monogynum* in the phylogenetic tree was shorter than that of *H. addingtonii*, with a support value of 100%, indicating that *H. monogynum* experienced more nucleotide variation than *H. addingtonii*, contrary to Nürk et al.’s phylogenetic analysis results for *H. monogynum* and *H. addingtonii*. This provides additional data for studying the complete phylogenetic relationships of *Hypericum*. This study used chloroplast genome data to achieve higher support values and resolution, providing more reliable evidence for elucidating the evolutionary history of *Hypericum* species.

### 3.4. Implications for Sustainable Agriculture and Medicinal Plant Conservation

This study’s findings have significant implications for sustainable agriculture and the conservation of medicinal *Hypericum* species. We identified highly variable regions in both coding and non-coding areas of the chloroplast genome, providing a comprehensive set of molecular tools for crop improvement and conservation efforts. In the LSC region, we found highly variable coding sequences (*accD*, *rpoC2*, *rpoB*) and non-coding sequences (*psbA-rbcL*, *trnV-UAC-trnL-UAA*, *trnG-GCC-psbZ*, *psbD-trnT-GGU*, *atpI-atpH*, *accD-trnN-GUU*). The SSC region also showed variable coding sequences (*matK*, *rpl32*, *rpl33*, *rps4*) and non-coding sequences (*matK-trnN-GUU*, *psaI-rpl32*, *rps15-ndhF*). These diverse markers can be utilized in marker-assisted selection programs to develop *Hypericum* varieties with enhanced medicinal properties or improved agricultural traits. For instance, the *accD* gene and its adjacent non-coding region (*accD-trnN-GUU*) could be targets for improving fatty acid biosynthesis and overall plant metabolism. The *rpoB* and *rpoC2* genes, along with intergenic regions like *psbA-rbcL*, might be explored for developing varieties with improved stress tolerance, a key factor in sustainable cultivation [52]. The various ribosomal protein genes and their associated intergenic regions provide multiple targets for investigating plant growth and development, potentially leading to more robust and productive *Hypericum* varieties.

The SSR and long-repeat sequence data, combined with the identified variable re-gions, offer powerful tools for genetic diversity assessment, crucial for both breeding programs and conservation strategies. By analyzing these markers across different *Hypericum* populations, we can gain comprehensive insights into the genetic structure and diversity of these species. This information is essential for identifying unique genetic resources and prioritizing populations for conservation. For example, populations with high diversity in both coding genes and intergenic regions could be given priority in conservation efforts to preserve maximum genetic variability. In breeding programs, these genetic diversity data can guide the selection of parental lines to maximize heterosis and develop varieties with improved traits. The intergenic regions, particularly those like *trnV-UAC-trnL-UAA* and *trnG-GCC-psbZ*, which are known to be highly variable in many plant species, could be especially useful for distinguishing closely related *Hypericum* species or populations.

The molecular tools developed in this study, including both coding and non-coding regions, have significant applications in the authentication of *Hypericum* species used in herbal products, contributing to sustainable use and conservation. Accurate identification is crucial in the medicinal plant industry to ensure product quality and efficacy. The highly variable regions identified, such as the *matK* gene and intergenic spacers like *psbD-trnT-GGU* and *atpI-atpH*, could serve as effective DNA barcodes for species authentication. This application extends beyond quality control in the herbal product industry; it can also aid in monitoring wild harvesting practices and prevent the overexploitation of rare *Hypericum* species. By providing a reliable means of species identification using both coding and non-coding markers, these molecular tools can support the development of sustainable wildcrafting guidelines and help balance conservation needs with the commercial use of *Hypericum* species [53]. Future research should focus on validating these markers across a wider range of *Hypericum* species and populations, developing user-friendly, cost-effective authentication protocols, and exploring how the diversity in these regions correlates with medicinal properties and environmental adaptations.

## 4. Materials and Methods

### 4.1. Sample Collection and DNA Extraction

Wild *Hypericum* plants (*Hypericum acmosepalum*, *Hypericum addingtonii*, and *Hypericum beanii*) were collected from the field in Kunming, Yunnan. The twigs and leaves of *H. acmosepalum*, *H. addingtonii*, and *H. beanii* were collected by Kunming Zhifen Biotechnology Company from Yunnan Province in June 2020. The authentication of the plant material was also carried out by the company. Fresh leaves from each of the three *Hypericum* species were harvested, sealed, and transported to the laboratory for genomic DNA extraction. The leaves were quickly frozen in liquid nitrogen and ground using a mortar and pestle. Genomic DNA was extracted from the pulverized samples using the Magen Plant DNA Extraction Kit. The concentration and purity of the DNA were evaluated using a NanoDrop 2000 spectrophotometer.

### 4.2. DNA Sequencing and Assembly

DNA sequencing was performed by Shanghai Yuanxin BioPharmaceutical Technology Co., Ltd. (Shanghai, China), using the Illumina NovaSeq6000 sequencing platform. The quality and integrity of the genomic DNA were initially assessed via 1% agarose gel electrophoresis. Qualified samples were then fragmented to suitable sizes by ultrasonic shearing, followed by end repair and purification to construct sequencing libraries for high-throughput sequencing. Base-calling analysis converted the raw image data files from sequencing into FASTQ format. Cutadapt V1.16 [54] was used to filter raw reads, removing low-quality sequences, adapter sequences, sequences with a high percentage of Ns (>10%), and sequences with a quality score below Q20. Quality control checks were performed using FastQC V0.11.4 [55] to obtain clean reads of acceptable quality. The clean reads were saved in FASTQ format for further analysis to ensure the reliability of the results.

De novo assembly of the chloroplast genomes was performed using three software tools: NOVOPlasty V4.2 [56], Fast-plast V1.2.8 [57], and GetOrganelle V1.7.0+ [58], with cross-validation to confirm the accuracy of the assembly. The most optimal assembly result was selected. The assembled chloroplast genomes were confirmed to be complete and circular.

### 4.3. Chloroplast Genome Annotation

Chloroplast genome gene prediction and annotation were conducted using tools such as PGA (https://github.com/quxiaojian/PGA) (accessed on 20 December 2023) [59] and GeSeq (https://chlorobox.mpimp-golm.mpg.de/geseq.html) (accessed on 20 December 2023) [60]. Manual correction of annotation results for all samples was performed using Notepad++ and Geneious [61]. Subsequently, annotated genome sequence files were submitted to OGDRAW (http://ogdraw.mpimp-golm.mpg.de) (accessed on 22 December 2023) [62] using default settings to establish the localization of inverted repeat sequences (IRs) relative to large single-copy (LSC) and small single-copy (SSC) regions, aiding in the creation of physical maps of the circular chloroplast genomes. The annotated chloroplast genome sequence files were submitted to the National Center for Biotechnology Information (NCBI) database (https://www.ncbi.nlm.nih.gov/) (accessed on 30 August 2024) to obtain genome accession numbers: *Hypericum acmosepalum* (SRR30615080), *Hypericum addingtonii* (SRR30615079), and *Hypericum beanie* (SRR30615078).

### 4.4. SSRs, Long Repeats, and Codon Usage Analysis

Simple sequence repeats (SSRs) in the chloroplast genomes of *Hypericum* species were analyzed using MISA (https://webblast.ipk-gatersleben.de/misa/) (accessed on 8 February 2024) [63], identifying the types, quantities, and loci of SSRs in the whole chloroplast genome sequences of the three *Hypericum* species. The analysis parameters were set as follows: the minimum repeat unit number for mononucleotides was set to 10, for dinucleotides to 6, and for trinucleotides, tetranucleotides, pentanucleotides, and hexanucleotides to 5. The maximum allowable interruption between SSRs was set to 100 base pairs (bp).

Long-repeat sequences, including palindromic, forward, reverse, and complementary repeats, were detected in the chloroplast genome sequences using the REPuter [64] online tool (https://bibiserv.cebitec.uni-bielefeld.de/reputer?id=reputer_manual_manual) (accessed on 8 February 2024) with the following parameters: Hamming distance of 3 and minimum repeat size of 30 bp. Additionally, relative synonymous codon usage (RSCU) of protein-coding genes was analyzed using CodonW (version 1.4.4, written by John Peden, http://sourceforge.net/projects/codonw) (accessed on 8 February 2024) [65] to study codon usage preferences of the target organisms.

### 4.5. Genome Comparison and Structural Analysis

Comparative maps of the IR boundary regions were created using IRScope (https://irScope.shinyapps.io/Irapp/) (accessed on 24 February 2024) [66] to quantitatively compare the gene features of each boundary region (LSC–IRa, Ira–SSC, SSC–IRb, IRb–LSC) and adjacent genes, providing an intuitive analysis of boundary changes in the evolutionary history of Hypericaceae species. The chloroplast genome sequences were aligned and visualized using the mVISTA [67] online tool (https://genome.lbl.gov/vista/mvista/submit.shtml) (accessed on 24 February 2024) in Shuffle-LAGAN global alignment mode with other parameters set to default to detect gene rearrangements and inversions within cp genomes.

### 4.6. Phylogenetic Analysis and Nucleotide Polymorphism Analysis

For the phylogenetic analysis of the sequenced *Hypericum* species, the chloroplast genomes of 14 Hypericaceae species and 2 *Populus* species (used as outgroups) were selected, with 13 species’ chloroplast genome sequences obtained from the NCBI database. Multiple sequence alignments of the 16 genomes were performed using MAFFT (v7.158b) [68]. Phylogenetic analysis was conducted using the maximum likelihood (ML) method based on single-copy genes (CDS) with RAxML v8.2.12 [69] to obtain the optimal ML tree.

To uncover the phylogenetic relationships among Hypericaceae species and the connection between chloroplast genome structure and species evolution, an online platform (https://www.timetree.org/) (accessed on 23 April 2024) was utilized to retrieve literature data from publication databases such as Google Scholar and PubMed, focusing on records of divergence times for Hypericaceae species. Based on these data, 10 Hypericaceae species and one outgroup plant were selected for the construction of a time-calibrated phylogenetic tree. TimeTree aggregates the previously reported times within the confidence intervals using the median value, minimizing the influence of outliers. It further adjusts the time nodes according to the divergence times of all direct descendants of the species, ensuring the credibility of the time nodes [70].

Additionally, nucleotide polymorphism analysis of the multiple sequence alignment results was performed using DnaSP v6.12 [71], and the results were plotted as a line graph.

## 5. Conclusions

In conclusion, this study reported for the first time the complete chloroplast genomes of *Hypericum acmosepalum*, *Hypericum addingtonii*, and *Hypericum beanii* and revealed the evolutionary characteristics of the chloroplast genomes of *Hypericum* and its closely related groups through comparative genomics analysis. The study found that the chloroplast genomes of these three plants exhibited significant differences in structural characteristics, gene content, and arrangement compared to other *Hypericum* species. The primary distinctions lie in the contraction and expansion of the inverted repeat (IR) regions, which alter the structure and gene distribution of the chloroplast genomes.

Analysis of long-repeat sequences and SSRs showed that *Hypericum* chloroplast genomes have rich polymorphism, especially in the mononucleotide repeats of the LSC region. These repeat sequences provide potential molecular markers for species identification and molecular breeding. This finding offers valuable genetic information for assessing the genetic diversity of wild *Hypericum* populations, enabling species identification for large-scale cultivation of medicinal *Hypericum* species and facilitating the development of improved *Hypericum* varieties.

Phylogenetic analysis and divergence time trees demonstrated the unique evolutionary characteristics of *Hypericum* species in terms of phylogenetic relationships and divergence times. The evolutionary affinities among *H. acmosepalum*, *H. addingtonii*, and *H. beanii* were analyzed. Comparative genomic analysis provides molecular data support for *Triadenum* as a genus that is phylogenetically close but independent from *Hypericum*, indicating the independent differentiation status of *Triadenum*.

This study provides important molecular marker resources for phylogenetic analysis, species identification, and genetic diversity studies of *Hypericum* and its closely related groups. It also offers new perspectives for further exploring the evolutionary mechanisms and functions of *Hypericum* chloroplast genomes. Furthermore, the findings of this research have significant implications for sustainable agriculture and medicinal plant conservation. The obtained molecular markers and genetic insights provide tools for molecular breeding to cultivate species with genes conferring resistance to environmental stress, thereby facilitating large-scale cultivation of medicinal *Hypericum* plants. Future research should focus on translating these genomic insights into practical applications for sustainable *Hypericum* cultivation and conservation.

## Figures and Tables

**Table 1:** Clean reads information of the three sequenced *Hypericum* species.

Species Name	Total_Reads	Total_Bases	Error%	Q20%	Q30%	GC%
*H. acmosepalum*	98,886,772	14,593,264,286	0.0244	98.26	94.68	40.14
*H. addingtonii*	86,191,406	12,706,259,381	0.0246	98.20	94.52	40.45
*H. beanii*	86,626,062	12,759,673,378	0.0242	98.36	94.90	39.53

Note: The unite of total bases is bp.

**Table 2:** Basic characteristics of the chloroplast genomes of 16 species.

Species Name	Total Length	LSC Length	SSC Length	IR Length	Coding Length	Non-Coding Length	Genes	Protein-Coding Genes	tRNA Genes	rRNA Genes	Pseudo Genes	GC Content	GC Content of LSC	GC Content of SSC	GC Content of IR
*Hypericum acmosepalum*	152,654	126,934	3044	11,338	65,820	86,834	127	86(10)	33(6)	8(4)	0	37.55	38.57	33.71	32.36
*Hypericum addingtonii*	122,570	98,190	9574	7403	53,751	68,819	112	75(0)	29(2)	8(4)	0	38.01	37.11	34.85	38.65
*Hypericum beanii*	137,652	99,212	10,988	13,726	57,861	79,791	116	75(2)	33(5)	8(4)	0	38.14	36.63	32.44	45.90
*Hypericum ascyron*	162,286	97,542	11,052	26,846	76,692	85,594	119	77(3)	34(6)	8(3)	8	37.36	36.46	32.33	40.04
*Hypericum monogynum*	138,972	93,123	11,081	17,385	57,732	81,240	119	77(1)	33(6)	8(4)	0	37.79	36.39	32.05	43.37
*Hypericum hookerianum*	138,260	120,848	10,950	3231	55,167	83,093	109	71(1)	34(6)	4(0)	0	38.08	38.80	32.58	34.01
*Hypericum perforatum*	139,725	95,109	11,084	16,766	61,263	78,462	119	74(2)	37(7)	8(4)	0	37.41	35.88	31.41	43.76
*Cratoxylum sumatranum*	157,089	86,029	18,760	26,150	77,676	79,413	128	83(6)	37(7)	8(4)	3	36.26	34.02	29.96	42.22
*Cratoxylum pruniflorum*	156,962	86,027	18,753	26,091	77,784	79,178	127	82(6)	37(7)	8(4)	4	36.27	33.97	29.95	42.32
*Cratoxylum maingayi*	157,358	86,033	18,971	26,177	78,075	79,283	128	83(6)	37(7)	8(4)	3	36.25	34.02	29.91	42.21
*Cratoxylum arborescens*	157,792	86,697	18,641	26,227	77,964	79,828	128	83(6)	37(7)	8(4)	3	36.15	33.80	29.95	42.22
*Cratoxylum formosum*	156,978	86,062	18,760	26,078	77,562	79,416	127	82(6)	37(7)	8(4)	3	36.26	34.02	29.95	42.21
*Cratoxylum cochinchinense*	157,103	85,664	18,895	26,272	79,014	78,089	128	83(7)	37(7)	8(4)	3	36.21	33.96	29.95	42.12
*Triadenum breviflorum*	167,693	85,268	11,185	35,620	83,931	83,762	128	83(8)	37(7)	8(4)	0	37.44	35.69	31.66	40.45
*Populus alba*	156,505	84,618	16,567	27,660	53,772	102,733	91	66(1)	23(6)	2	2	36.74	34.56	30.49	41.95
*Populus wulianensis*	156,553	84,726	16,551	27,638	80,697	75,856	129	84(8)	37(7)	8(4)	0	36.72	34.51	30.53	41.96

Note: The unite of genome length is bp.

## Data Availability

The datasets presented in this study can be found in Supplementary Materials.

## Supplementary Materials

The supporting information can be downloaded at https://www.mdpi.com/article/10.3390/ijms26010323/s1.
